# Identifying sources of variation in parasite aggregation

**DOI:** 10.7717/peerj.13763

**Published:** 2022-08-24

**Authors:** André Morrill, Ólafur K. Nielsen, Karl Skírnisson, Mark R. Forbes

**Affiliations:** 1 Biology Department, Carleton University, Ottawa, Ontario, Canada; 2 Icelandic Institute of Natural History, Garðabær, Iceland; 3 Institute for Experimental Pathology, Keldur, University of Iceland, Reykjavík, Iceland

**Keywords:** Aggregation, Bayesian analysis, *Lagopus muta*, Parasitism, Rock ptarmigan

## Abstract

Aggregation of macroparasites among hosts is a near-universal pattern, and has important consequences for the stability of host-parasite associations and the impacts of disease. Identifying which potential drivers are contributing to levels of aggregation observed in parasite-host associations is challenging, particularly for observational studies. We apply beta regressions in a Bayesian framework to determine predictors of aggregation, quantified using Poulin’s index of discrepancy (*D*), for 13 species of parasites infecting Icelandic Rock Ptarmigan (*Lagopus muta*) collected over 12 years. 1,140 ptarmigan were collected using sampling protocols maximizing consistency of sample sizes and of composition of host ages and sexes represented across years from 2006–2017. Parasite species, taxonomic group (insect, mite, coccidian, or nematode), and whether the parasite was an ecto- or endoparasite were tested as predictors of aggregation, either alone or by modulating an effect of parasite mean abundance on *D*. Parasite species was an important predictor of aggregation in models. Despite variation in *D* across samples and years, relatively consistent aggregation was demonstrated for each specific host-parasite association, but not for broader taxonomic groups, after taking sample mean abundance into account. Furthermore, sample mean abundance was consistently and inversely related to aggregation among the nine ectoparasites, however no relationship between mean abundance and aggregation was observed among the four endoparasites. We discuss sources of variation in observed aggregation, sources both statistical and biological in nature, and show that aggregation is predictable, and distinguishable, among infecting species. We propose explanations for observed patterns and call for the review and re-analysis of parasite and other symbiont distributions using beta regression to identify important drivers of aggregation—both broad and association-specific.

## Introduction

Macroparasites (hereafter parasites) are often aggregated on their hosts, whereby many host individuals have few or no parasites and relatively few hosts harbor higher-intensity infections (Crofton, 1971; Shaw & Dobson, 1995). Parasite aggregation is such a common pattern in nature that it has been dubbed a law of parasite ecology (Poulin, 2007a). Parasite aggregation is an important law given that parasites are expected to show intensity-dependent effects on their hosts, such as reductions in host fecundity and longevity (Gleave et al., 2016; Hudson, Newborn & Dobson, 1992; Shostak, Van Buuren & Cook, 2015). Furthermore, degree of parasite aggregation influences likelihood of those fitness impacts scaling up to host populations (Hudson, Newborn & Dobson, 1992).

Despite being a law, parasite aggregation still shows considerable variation from moderate to extreme cases of aggregation (Johnson & Hoverman, 2014; Poulin, 2007b; Poulin, 2013), likely due to variation in the type or strength of its underlying causes (Poulin, 2007b). The search for biological predictors of parasite aggregation must recognize that aggregation is a phenomenon of both the parasite and host species. Factors promoting aggregation such as reproduction and recruitment of parasites on hosts (Grear & Hudson, 2011), attraction of infective stages of parasites to one another and environmental aggregation (Morrill & Forbes, 2016), and host condition-linked immunity (Morrill & Forbes, 2012) must be considered alongside factors reducing aggregation such as density-dependent mortality of parasites (Anderson & May, 1978; Luong, Vigliotti & Hudson, 2011) and parasite-induced host mortality (Anderson & Gordon, 1982). Such factors can be variably important in determining the degree of parasite aggregation observed, both within and across species associations.

On the one hand, differences in underlying mechanisms producing or minimizing aggregation in host-parasite associations may promote differences in observed levels of aggregation. They may also promote differences in relations of indices of aggregation to other parasitological measures such as prevalence (the proportion of sampled individuals infected by one or more of the focal parasite) or mean abundance (the average number of focal parasites infecting any host). For example, acquired immunity to parasites operating in one parasite-host association, and the frequency of which sampled individuals show that acquired immunity (Luong, Vigliotti & Hudson, 2011), may affect both variation in mean parasite abundance and aggregation across samples. Very different patterns of aggregation (and mean abundance) are expected, all else being equal, if hosts were to altogether lack the ability to acquire immunity to parasites. On the other hand, host-symbiont associations with similar degrees of aggregation need not share the same or similar mechanisms of aggregation. As a first approximation of the diversity of (dis)aggregative mechanisms operating in nature, researchers can test whether different (groups of) associations differ predictably in their levels of aggregation, or in relations of aggregation to other measures of infection. Researchers can also group parasite-host associations by parasite and/or host traits and see if these traits are predictive of aggregation or its relations.

Icelandic Rock Ptarmigan (*Lagopus muta*, hereafter ptarmigan) are an ideal host species to study parasite aggregation because they have been subject to intense research as a game bird and have provided large numbers of replicates for ecological studies (Morrill et al., 2021; Nielsen et al., 2020; Stenkewitz et al., 2016); additionally, their parasite fauna are exceptionally well known and can be sampled with standardized protocols (Skírnisson, Thorarinsdottir & Nielsen, 2012; Stenkewitz et al., 2016). Parasites infecting ptarmigan from a focal population in northeast Iceland comprise a diverse set of ecto- and endoparasites, for which prevalence of several species correlates with host health indices and/or host population densities (Stenkewitz et al., 2016). Substantial variation in parasitological measures across years and between infecting species—species representing several broad taxonomic groups (insects, mites, coccidian protozoans, and nematodes)—permits the elucidation of potentially important predictors of aggregation in this host system.

There are many ways to measure parasite aggregation (see Poulin, 2007b; McVinish & Lester, 2020; Wilson et al., 2002 for reviews). One measure, Poulin’s *D* (Poulin, 1993), is the measure we adopt here in a comparison of aggregation within and between 13 species of symbionts typically viewed as parasites of ptarmigan. Poulin’s *D* is an index of discrepancy between two parasite accumulation curves: one based on observed data for a given parasite species and one wherein all hosts are infected by the same mean number of parasites of that species (Poulin, 1993). Thus, Poulin’s *D* theoretically ranges from zero to one (one representing the discrepancy between an even distribution of parasites *versus* all sampled parasites infecting a single host). This bounding between zero and one lends Poulin’s *D* to comparisons of aggregation between species associations (Wilson et al., 2002). Furthermore, Poulin’s *D* has the advantage over the oft-used index of aggregation of the negative binomial distribution, the dispersion parameter *k*, in that *D* can be calculated for any aggregated distribution, and not just for those following a negative binomial. Poulin’s *D* can be used instead of variance-to-mean ratios or Taylor Power Law approaches, which are sensitive to the tail of the distribution (Scott, 1987; but see Johnson & Wilber, 2017). One other measure, Hoover’s index, is similarly bounded between zero and one and has the advantage of being interpretable as the proportion of parasites that would have to be re-assigned to achieve an even parasite distribution (McVinish & Lester, 2020). However, Hoover’s index is tightly correlated with Poulin’s *D* due to both measures deriving from comparisons of cumulative infection curves (wherein parasite loads are ordered from lowest to highest intensity; analogous to Lorenz curves), and a theoretical even distribution of the same total number of infecting parasites (McVinish & Lester, 2020). Therefore, use of Poulin’s D and Hoover’s index are unlikely to produce different major results.

We used a unique data set based on 13 parasite associations of ptarmigan, reported over 9–12 years in northeast Iceland. We applied a Bayesian approach based on beta regression with Poulin’s *D* as the dependent variable, to address the following related questions: first, do parasite-host associations show consistency in their degree of aggregation across samples (years)—*i.e.,* are there parasite species-specific degrees of aggregation (formally tested by evaluating model support for separate intercepts for each parasite species)? Second, are there any expected relationships between Poulin’s *D* and mean abundance across samples for given parasite-host associations, and do these differ between associations (formally tested by evaluating model support for one or more slope terms)? Third, does parasite taxonomic grouping (insect, mite, coccidian, nematode) or parasite type (ectoparasite *vs.* endoparasite) account for any variation in either degree of aggregation or its relation to mean abundance?

We show species-specific degrees of aggregation that cannot be grouped on broader taxonomic grounds, nor can they be grouped based on the endoparasite *versus* ectoparasite dichotomy. We further show apparent dependence of degree of aggregation (Poulin’s *D*) on mean abundance of symbionts in samples for nine of 13 associations, but whereas these patterns cannot be grouped taxonomically, they group differently for symbionts typically considered ectoparasites (but which include paraphages) *versus* endoparasites (inverse relations *versus* no relations, respectively). We explore possible statistical and biological determinants of degrees of aggregation and its relation to mean abundance across associations. We also call for a re-analysis of existing data using the beta regression technique and Bayesian inference to uncover the determinants of degrees of parasite aggregation in other associations. Such analyses will help address the extent to which parasite (and symbiont) aggregation represents a diversity of patterns with diverse explanations *versus* fewer patterns with fewer well represented (dis)aggregative mechanisms operating. We thus propose and formalize a statistical approach to the study of predictors of parasite aggregation, and describe and explain variation in aggregation in an assemblage of symbionts of ptarmigan.

## Materials & Methods

### Sample collection and the symbiont assemblage

Samples of 79–102 ptarmigan were collected by hunting in each autumn of 12 years (collections organized by the Icelandic Institute of Natural History) to ensure sufficient sample size for reliable estimates of infection measures (Wilson et al., 2002). Furthermore, *ca.* 20 adult male, 20 adult female, and 60 juveniles (30 of both sexes) were collected each year which minimized any effects of differential representation of age or sex classes of birds between years on infection measures (see Nielsen et al., 2020). In eight of 12 years, sample size was at least 100. Processing of samples and standardized protocols to enumerate parasites are detailed elsewhere (Skírnisson, Thorarinsdottir & Nielsen, 2012; Stenkewitz et al., 2016). However, some salient points bear repeating. Only parasites identifiable to species and that were not rare (<2% prevalence) were included in analyses. In total, our study included four taxa of symbionts, comprising 13 species: four insects (three lice and one fly), five mites, two species of coccidians, and two nematodes.

All parasite species were observed in all years, except for *Trichostrongylus tenuis* which was absent in 2007, 2008, and 2012, and *Mironovia lagopus*, for which there were no abundance measures greater than zero in 2006 (though this mite was still noted as present in some feathers in this year, it was not represented in samples based on filtered vacuuming).

The coccidians included the specialist *Eimeria muta* whose annual prevalence was shown to be correlated with time-lagged population cycles of ptarmigan (Stenkewitz et al., 2016), and the less common *E. rjupa*. Coccidians are often considered microparasites because they multiply within their hosts during the endogenous phase of their life cycle, though elsewhere they may be grouped with macroparasites because they cause intensity-dependent pathology (Norton, 1967; Trigg, 1967). The two other endoparasites were nematodes—*Capillaria caudinflata* and *Trichostrongylus tenuis*—which live in the small intestine and ceca, respectively. *C. caudinflata* is associated with overwintering mortality of juvenile birds (Stenkewitz et al., 2016), but the far less abundant *T. tenuis* was not associated with serious disease outcomes in these ptarmigan (Stenkewitz et al., 2016). In other studies, *T. tenuis* is much more prevalent and numerous in wild grouse and it is inversely related to fecundity (*e.g.*, Dobson & Hudson, 1992).

The ectoparasites included skin mites *Metamicrolichus islandicus* (known to cause mange) and *Myialges borealis*, the two often appearing in co-infections (Stenkewitz et al., 2015). The quill mite (*Mironovia lagopus*), another ectoparasite, was infrequently sampled, whereas the two remaining mite species *Strelkoviacarus holoapsis* and the wing mite *Tetraolichus lagopi* were often sampled on birds (*T. lagopi* nearly always showed 100% prevalence in samples). These two species are suspected of being paraphages or mutualistic symbionts rather than true parasites (Stenkewitz, 2017; Doña et al., 2019). The three ectoparasitic lice included the chewing louse *Amyrsidea lagopi* which is thought to be harmful to birds by damaging plumage (Stenkewitz et al., 2016) and the highly specialized *Goniodes lagopi* and *Lagopoecus affinis* which feed on keratin. The fourth insect species, the hippoboscid fly *Ornithomya chloropus,* is an ectoparasite that sucks blood from its bird host and which acts as a vector of mites of ptarmigan (Guðmundsson, Skírnisson & Nielsen, 2021).

### Statistical methods

Our chosen measure for quantifying aggregation, Poulin’s *D*, is calculated as:

(1) \begin{eqnarray*}D=1- \frac{2\sum _{i=1}^{N}(\sum _{j=1}^{i}{x}_{j})}{\bar {x}N(N+1)} \end{eqnarray*}

where *N* is the total number of hosts in the sample, $\bar {x}$ is the average number of parasites, and *x* is the number of parasites infecting host *j*, with hosts ordered from least to most heavily infected (Poulin, 1993). *D* is thus constrained to fall between approximately zero (minimum aggregation; an even distribution of parasites) and one (maximum aggregation; all parasites infecting a single host) regardless of parasite mean abundance. The measure therefore lends itself to analysis of aggregation across samples, studies, and/or species.

Modeling was conducted in a Bayesian framework using Stan software (Stan Development Team, 2019), which implements an adaptive method of Hamiltonian Monte Carlo (HMC) sampling of posterior distributions *via* the No-U-Turn sampler (NUTS; Hoffman & Gelman, 2014; Stan Development Team, 2019). The package ‘cmdstanr’ (Gabry & Cešnovar, 2021) was used as an interface between Stan and the statistical programming language R (Version 3.6.1; R Core Team, 2019).

We modeled *D* using beta regressions implemented as generalized linear mixed models (GLMMs; beta-distributed response, with a logit link function) because *D* is constrained between zero and one. Use of beta regression to model parasite aggregation appears unprecedented but provides an effective tool for assessing determinants/correlates of aggregation. Additionally, the ease of implementing custom hierarchical model structure afforded by Bayesian modeling when using software like Stan also allows effective information “sharing” across predictor factor levels through the incorporation of random effects, and the specification of even weakly-informative priors may permit the fitting of otherwise prohibitively complex, though biologically justified, models. A Bayesian approach could directly incorporate uncertainty in the response and/or predictors, a further benefit to analyzing predictors of aggregation across studies. We nonetheless recommend beta regression as an effective tool for modeling aggregation as measured by *D* regardless of whether the implementation is Bayesian, as it is here, or not.

Beta distributions are generally described by two parameters, though there are different choices of parameterizations. Here, we used the mean (µ) and variance (*ν*) parameterization rather than the more common *α* and *β* parameterization (to switch between the two: *α* = µ*ν*, and *β* = (1 − µ) *ν*). This choice allowed us to directly estimate mean values for *D* between zero and one, following the linear model. When *ν* is lower than two, the probability density of the beta distribution begins getting “pushed” towards the extremes of zero and one, rather than being spread (diminishing) around the mean (McElreath, 2020). Therefore, the implemented beta regression sets a lower limit of two on the variance parameter *ν via* a half-Cauchy prior to ensure that modeled aggregation levels are concentrated around the means (McElreath, 2020).

The predictors of aggregation in fitted models included at least one intercept term, possible slope terms relating sample mean abundance to aggregation, and a random effect of sampling year. Often, mean abundance is expected to relate negatively to aggregation, including when measured using Poulin’s *D* (Poulin, 1993; Shaw, Grenfell & Dobson, 1998; Johnson & Wilber, 2017). Mean abundance was log_2_-transformed due to high overall positive skew, and then centered and standardized before modeling. In addition to a single-intercept (null) model, we considered candidate models with intercept (*θ*) terms indexed either by parasite species, taxonomic grouping (insect, mite, coccidian, nematode), or the ectoparasite-endoparasite dichotomy. The potential mean abundance relationship, added to relevant models as a slope term (*β*), was either included as a single parameter or indexed by the same potential groupings as listed above (*i.e.,* species, broader taxonomic grouping, or ecto-/endoparasite). Therefore, we compared models to test whether parasite biology, either on its own or through its modulation of potential aggregation-mean abundance relationships, was an important predictor of aggregation. A single intercept, single slope candidate model follows the form:

\begin{eqnarray*}{D}_{i}\sim \text{Beta} \left( {\mu }_{i},\nu \right) \end{eqnarray*}

\begin{eqnarray*}\text{logit}({\mu }_{i})=\theta +\beta \times {m}_{i}+{\lambda }_{YEAR[i]} \end{eqnarray*}

where *m*_*i*_ is the mean abundance of sample *i*, and *λ*_*YEAR*[*i*]_ is the random effect of the relevant year. Selected weakly-informative priors for the *θ*, *β*, *ν*, and *λ* parameters were:

\begin{eqnarray*}\theta \sim \text{Normal} \left( 0,1.5 \right) \end{eqnarray*}

\begin{eqnarray*}\beta \sim \text{Normal} \left( 0,2.2 \right) \end{eqnarray*}

\begin{eqnarray*}\nu \sim \text{Half-Cauchy} \left( 2,2.5 \right) \end{eqnarray*}

${\lambda }_{j}\sim \text{Normal} \left( 0,{\sigma }_{\lambda } \right) $ for *j* = 1..12

\begin{eqnarray*}{\sigma }_{\lambda }\sim \text{Exponential}(1) \end{eqnarray*}

Here, the notation Normal(*x, y*) refers to a normal distribution with mean *x* and standard deviation *y*; Half-Cauchy(*a*, *b*) refers to a half-Cauchy distribution with location parameter (and lower limit) *a* and scale parameter *b*; and Exponential(*c*) refers to an exponential distribution with rate parameter *c* (mean = 1/*c*). Prior predictive simulations exploring the relevance of the selected intercept and slope priors are presented in Article S1. The exponential prior with rate parameter equal to one on the standard deviation term follows McElreath (2020).

When the intercept or slope parameters were indexed by factors with more than two groups (*i.e.,* either parasite species or broader parasite taxonomic grouping), these were also included as random effects. For example, the linear model and *θ* and *β* priors above would, in the candidate species-specific intercept and group-specific slope model, instead become:

\begin{eqnarray*}\text{logit}({\mu }_{i})={\theta }_{SPECIES[i]}+{\beta }_{GROUP[i]}\times {m}_{i}+{\lambda }_{YEAR[i]} \end{eqnarray*}

${\theta }_{k}\sim \text{Normal} \left( \theta ,{\sigma }_{\theta } \right) $    for *k* = 1..13

${\beta }_{n}\sim \text{Normal} \left( \beta ,{\sigma }_{\beta } \right) $    for *n* = 1..4

\begin{eqnarray*}\theta \sim \text{Normal} \left( 0,1.5 \right) \end{eqnarray*}

\begin{eqnarray*}\beta \sim \text{Normal} \left( 0,2.2 \right) \end{eqnarray*}

\begin{eqnarray*}{\sigma }_{\theta }\sim \text{Exponential}(1) \end{eqnarray*}

\begin{eqnarray*}{\sigma }_{\beta }\sim \text{Exponential}(1) \end{eqnarray*}

It is possible that intercept and slope parameters of a linear model may covary when indexed by the same grouping variable: higher intercepts may relate to higher—or lower—slope terms. Therefore, whenever *θ* and *β* random effects were indexed by the same variable, these were both modeled as following a single multivariate normal distribution, as this model structure allows underlying correlation to be incorporated, and to inform parameter estimation (McElreath, 2020). The correlation matrix for *θ* and *β*, ***R***, can be factored out of the multivariate normal distribution covariance matrix, ***S***, resulting in this formulation (for the sake of example, given the model wherein both intercepts and slopes are species-specific):

\begin{eqnarray*}{D}_{i}\sim \text{Beta} \left( {\mu }_{i},\nu \right) \end{eqnarray*}

\begin{eqnarray*}\text{logit}({\mu }_{i})={\theta }_{SPECIES[i]}+{\beta }_{SPECIES[i]}\times {m}_{i}+{\lambda }_{YEAR[i]} \end{eqnarray*}

\begin{eqnarray*} \left[ \begin{array}{@{}c@{}} \displaystyle {\theta }_{k}\\ \displaystyle {\beta }_{k} \end{array} \right] \sim \text{MVNormal} \left( \left[ \begin{array}{@{}c@{}} \displaystyle \bar {\theta }\\ \displaystyle \bar {\beta } \end{array} \right] ,S \right) \end{eqnarray*}

\begin{eqnarray*}S= \left( \begin{array}{@{}cc@{}} \displaystyle {\sigma }_{\theta }&\displaystyle 0\\ \displaystyle 0&\displaystyle {\sigma }_{\beta } \end{array} \right) R \left( \begin{array}{@{}cc@{}} \displaystyle {\sigma }_{\theta }&\displaystyle 0\\ \displaystyle 0&\displaystyle {\sigma }_{\beta } \end{array} \right) \end{eqnarray*}

\begin{eqnarray*}\bar {\theta }\sim \text{Normal} \left( 0,1.5 \right) \end{eqnarray*}

\begin{eqnarray*}\bar {\beta }\sim \text{Normal} \left( 0,2.2 \right) \end{eqnarray*}

\begin{eqnarray*}\nu \sim \text{Half-Cauchy} \left( 2,2.5 \right) \end{eqnarray*}

\begin{eqnarray*}{\lambda }_{j}\sim \text{Normal} \left( 0,{\sigma }_{\lambda } \right) \end{eqnarray*}

\begin{eqnarray*}{\sigma }_{\theta }\sim \text{Exponential}(1) \end{eqnarray*}

\begin{eqnarray*}{\sigma }_{\beta }\sim \text{Exponential}(1) \end{eqnarray*}

\begin{eqnarray*}{\sigma }_{\lambda }\sim \text{Exponential}(1) \end{eqnarray*}

\begin{eqnarray*}R\sim \text{LKJcorr}\text{(2)}. \end{eqnarray*}

The weakly-informative prior on the correlation matrix ***R*** is the Lewandowski-Kurowicka-Joe distribution, with its *η* parameter set to two, as this prior is skeptical of extreme correlations (*i.e.,* those close to −1 or 1), and has its probability density centred around zero correlation (Lewandowski, Kurowicka & Joe, 2009; Stan Development Team, 2019).

We fit each Bayesian model using three Markov chains, with 2,500 warm-up iterations and 1,500 sampling iterations (total samples = 3 × 1,500 = 4,500). Effective sample sizes for estimated parameters were assessed to ensure accurate estimation (ESS >100 per chain; Vehtari et al., 2021), and *Rˆ* convergence diagnostics for all parameters were checked to ensure they were less than 1.01 (an indication that chains had mixed well). The R package ‘shinystan’ was used to help assess model convergence (Gabry, 2018), and functions from the package ‘tidybayes’ provided tools for exploring posterior distributions (Kay, 2021). Posterior predictive checks were conducted to ensure that model estimates were consistent with observed data.

All fitted models were compared by their estimated out-of-sample predictive performance during leave-one-out cross validation, based on estimated log pointwise predictive density (ELPD; higher values indicate more predictive models). This method considers the entire posterior distribution, and relies on Pareto-smoothed importance sampling (PSIS) to calculate estimates (Vehtari, Gelman & Gabry, 2017). ELPD estimates, as well as the estimates of the difference in ELPD between a given model and the overall best-performing model, are provided with calculated standard errors, allowing the assessment of levels of confidence (*e.g.*, if a model has higher ELPD compared to another, but the standard error in that estimated difference is larger than the difference itself, then there is very little confidence that the two models differ meaningfully in out-of-sample predictive performance). Additionally, PSIS calculations provide Pareto *k* values for each observation, which can indicate when ELPD estimates are unreliable, and/or that particular observations have disproportionate influence on the posterior distribution; *k* values should be less than 0.7 (Vehtari, Gelman & Gabry, 2017). The ‘loo’ package provided functions for the calculation of ELPDs and their comparisons (Vehtari et al., 2020). All reported credible intervals (CIs) are 95% highest density continuous intervals.

## Results

**Figure 1:**
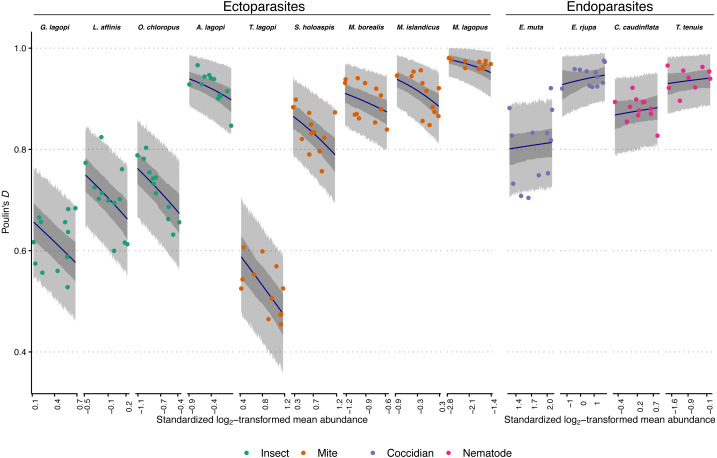
Observed aggregation levels of Rock Ptarmigan parasites, compared to best-fitting model predictions. The best-fitting model is a beta regression with species-specific intercepts and ecto-/endoparasite specific slopes relating aggregation, as measured using Poulin’s *D*, to (transformed) sample mean abundance. Coloured points represent observed aggregation levels for individual parasite species in given years (2006–2017). Dark blue lines represent model predictions of mean aggregation levels, while the dark grey regions are 95% credibility intervals for those means, calculated as highest density continuous intervals (HDCIs). The lighter grey regions are the 95% prediction intervals, also as HDCIs. Note that the ranges along the X-axes for each species are scaled differently to match the ranges of observed mean abundances. Parasite species are ordered from lowest to highest overall average aggregation level within each taxonomic grouping (insect, mite, coccidian, nematode). See Table 1 for full parasite species names.

Aggregation levels within years were generally high across parasite species (Table 1; overall average of within-year *D* measures = 0.82, sd = 0.14), and ranged from 0.46 to 0.98. Overall, 75% of within-year aggregation levels were greater than 0.71, and 50% were greater than 0.87. The only four *D* values less than 0.50 occurred exclusively in a species of paraphage, *T. lagopi*. While aggregation levels varied across a wide range, between-year *D* values showed some consistency within species (as described below, and visualized in Fig. 1; also, see Table 1).

**Table 1:** Overall parasite aggregation (all hosts across all years combined) and range of aggregation levels measured each year for each species, quantified using Poulin’s *D*. The difference between the maximum and minimum within-year aggregation levels is also provided. All parasites were observed in all years, except *T. tenuis* (present in nine of 12 years) and *M. lagopus* (no abundance measures greater than zero in 2006). 95% confidence intervals are bias-corrected and accelerated bootstrap intervals.

Parasite group	Parasite	Overall *D* (95% CI)	Range of *D* across years	Difference between max. and min. *D*
Coccidian	*Eimeria muta*	0.849 (0.807–0.891)	0.704–0.921	0.217
	*Eimeria rjupa*	0.978 (0.964–0.986)	0.92–0.975	0.055
Insect	*Amyrsidea lagopi*	0.935 (0.925–0.945)	0.847–0.966	0.119
	*Goniodes lagopi*	0.656 (0.636–0.676)	0.528–0.684	0.156
	*Lagopoecus affinis*	0.724 (0.704–0.744)	0.6–0.824	0.225
	*Ornithomya chloropus*	0.742 (0.719–0.761)	0.632–0.803	0.172
Mite	*Myialges borealis*	0.914 (0.902–0.928)	0.839–0.941	0.101
	*Metamicrolichus islandicus*	0.928 (0.915–0.939)	0.848–0.956	0.108
	*Mironovia lagopus*	0.985 (0.979–0.99)	0.959–0.98	0.022
	*Strelkoviacarus holoaspis*	0.861 (0.843–0.883)	0.757–0.898	0.142
	*Tetraolichus lagopi*	0.575 (0.558–0.596)	0.455–0.607	0.152
Nematode	*Capillaria caudinflata*	0.904 (0.891–0.917)	0.827–0.922	0.095
	*Trichostrongylus tenuis*	0.978 (0.969–0.985)	0.896–0.966	0.069

The beta regression GLMMs all fit without indications of convergence issues. Effective sample sizes were above 300 and $\hat {R}$ statistics were <1.01 for all parameters in all models, except for the standard deviation parameter for the underlying distribution of the random effect of year in most models. This was likely due to the effects of each year on aggregation never differing from zero (that is, there was no meaningful consistent effect of any year on aggregation of parasites across species). The algorithm thus had difficulty in estimating underlying variation in that random effect. However, effective sample sizes for this standard deviation parameter were still always greater than 152, and the $\hat {R}$ statistics were never more than 1.02, suggesting that the model formulations were not poor, and the models themselves still converged without issue. All Pareto-*k* values for all observations in all models were less than 0.7, indicating that no observations were particularly “surprising” given the candidate model specifications, and that PSIS ELPD estimates are likely reliable.

All models performed better than the null based on estimated out-of-sample predictive performance *via* ELPD (Table 2). The overall best model had species-specific intercepts and an ecto-/endoparasite-specific slope relating aggregation to sample mean abundance. Incorporating species-specific slopes or slopes based on broader taxonomic groupings did not change predictive performance; differences between these more highly parameterized candidate models and the top-scoring model were associated with standard errors larger than the differences themselves (Table 2). There was, however, a difference between the top-scoring model and the simpler single slope candidate model, suggesting that effects of mean abundance on aggregation were not equal between the ecto- and endoparasites (the difference in ELPD was more than twice as large as that difference’s associated standard error). Therefore, the two-slope parameter model (species-specific intercepts, ecto-/endoparasite-specific slopes) was chosen as the overall best-fitting, and most parsimonious model. Looking at the ELPD scores in Table 2, there is a large jump in predictive performance once model intercepts are indexed by species rather than any other grouping variable, further supporting species being important in predicting aggregation level, even after taking mean abundance into account.

**Table 2:** Beta regression models explaining aggregation of Rock Ptarmigan parasites as measured using Poulin’s *D*. Models are compared via Pareto-smoothed importance sampling (PSIS) estimates of out-of sample predictive performance during leave-one-out cross validation (estimated log pointwise predictive density; ELPD). Models are ordered from highest to lowest ELPD (most to least predictive). The difference in ELPD between any focal model and the overall best performing model is provided, as well as the effective numbers of model parameters. All numeric estimates are presented with standard errors.

Model	ELPD (SE)	Difference (SE)	Eff. params. (SE)
Species-specific intercept, endo/ecto-specific slope	283.1 (10.3)	0 (0)	17.7 (2.2)
Species-specific intercept, group-specific slope	282.4 (10.2)	−0.8 (1.3)	19.1 (2.1)
Species-specific intercept, species-specific slope	281.2 (10.5)	−2.0 (2.5)	24.2 (3.0)
Species-specific intercept, single slope	273.2 (10.5)	−10.0 (4.5)	17.5 (2.3)
Species-specific intercept	272.0 (10.1)	−11.1 (4.8)	18.0 (2.2)
Group-specific intercept, species-specific slope	227.5 (11.4)	−55.6 (9.7)	17.0 (2.1)
Endo/ecto-specific intercept, species-specific slope	204.4 (10.1)	−78.7 (9.7)	13.2 (1.8)
Single intercept, species-specific slope	184.9 (10.7)	−98.2 (10.9)	12.0 (1.7)
Group-specific intercept, endo/ecto-specific slope	165.9 (8.6)	−117.2 (10.3)	7.2 (0.8)
Group-specific intercept, group-specific slope	164.5 (8.7)	−118.6 (10.4)	8.1 (0.9)
Group-specific intercept, single slope	162.9 (8.8)	−120.2 (10.5)	7.0 (0.8)
Endo/ecto-specific intercept, endo/ecto-specific slope	157.1 (8.3)	−126.0 (9.7)	4.7 (0.6)
Endo/ecto-specific intercept, group-specific slope	155.6 (8.1)	−127.5 (9.6)	6.1 (0.7)
Endo/ecto-specific intercept, single slope	149.8 (7.5)	−133.3 (9.6)	4.6 (0.6)
Single intercept, group-specific slope	140.8 (7.9)	−142.4 (9.7)	5.6 (0.5)
Single intercept, endo/ecto-specific slope	137.7 (8.0)	−145.4 (10.1)	4.5 (0.5)
Single intercept, single slope	124.3 (9.0)	−158.8 (10.6)	3.4 (0.6)
Group-specific intercept	124.0 (9.8)	−159.1 (12.0)	4.9 (0.5)
Endo/ecto-specific intercept	118.5 (8.1)	−164.6 (10.8)	3.3 (0.3)
Null model (single intercept only)	114.8 (8.6)	−168.3 (10.9)	1.5 (0.2)

The best-fitting model parameter estimates are summarized in Table 3, and model predictions compared to observed data is visualized in Fig. 1. Figure 1 additionally demonstrates that the model posterior predictions fit the observed data well. Species intercepts (*θ* estimates) show a good deal of variation between species, and together these estimates and the distributions/trends in Fig. 1 demonstrate that parasite species is predictive of level of aggregation in any year for this population of Rock Ptarmigan. Average species-level aggregation was inconsistent within the higher taxonomic groupings; for example, the highest mean *θ* estimates among both insect and mite parasites were more than three times greater than the lowest mean estimates within those same groups, and the overall highest and lowest average *D* values for individual species were both associated with mites.

**Table 3:** Summary of parameter estimates from the selected best-fitting model. The selected model is that with species-specific intercepts and ecto/endoparasite-specific slopes relating mean abundance to parasite aggregation, measured using Poulin’s *D*. 95% credible intervals (CIs) are highest density continuous intervals. The estimated nu (*ν*) parameter is the variance of the beta-distributed response. Mean aggregation estimates predicted by this model must be inverse-logit-transformed to arrive back at the original scale of Poulin’s *D*. Rˆ-statistics for all estimated parameters were below 1.01, supporting the conclusion that Markov chains had converged.

Parameter		Mean estimate	95% CI	Effective sample size
theta (Intercept)	*Amyrsidea lagopi*	2.23	(1.99–2.48)	3,223.22
	*Capillaria caudinflata*	1.95	(1.76–2.15)	4,514.07
	*Eimeria muta*	1.28	(0.94–1.65)	2,141.40
	*Eimeria rjupa*	2.73	(2.45–3.02)	3,708.15
	*Goniodes lagopi*	0.73	(0.57–0.89)	2,510.79
	*Lagopoecus affinis*	0.82	(0.68–0.96)	4,618.68
	*Myialges borealis*	1.61	(1.33–1.87)	2,136.13
	*Metamicrolichus islandicus*	2.23	(2.03–2.44)	4,821.32
	*Mironovia lagopus*	2.16	(1.68–2.66)	1,808.90
	*Ornithomya chloropus*	0.51	(0.31–0.74)	1,721.42
	*Strelkoviacarus holoaspis*	2.04	(1.82–2.27)	2,088.89
	*Tetraolichus lagopi*	0.62	(0.40–0.86)	1,699.50
	*Trichostrongylus tenuis*	2.79	(2.47–3.12)	3,333.53
beta (Slope)	Ectoparasite	−0.6	(−0.82–−0.39)	1,351.64
	Endoparasite	0.10	(−0.10–0.27)	2,066.37
nu (Variance parameter)		84.07	(65.00–105.54)	4,397.93

The slope (*β*) parameters differed between ecto- and endoparasites: aggregation decreases with mean abundance similarly among the insects and mites, while no strong effect is seen for the coccidians and nematodes. Table 3 shows how the 95% credible interval around the *β* estimate for endoparasites is highly compatible with zero; *i.e.,* there is no evidence for decreasing (or increasing) aggregation with increasing mean abundance within this group. The ectoparasite-specific slope, however, is distinctly negative, and contrasts between the two *β* estimates within each Markov chain sample showed a distribution that excluded zero, further supporting a difference in the effect of mean abundance between the two groups (average difference between *β* estimates = 0.70; 95% CI [0.41–0.97]).

In summary, variation in *D* is explained by the species of associated parasites/symbionts and sample mean abundance, but not by higher taxonomic groupings. Additionally, whether the species was an ectoparasite/symbiont *versus* an endoparasite explained additional variation in *D*, through its interaction with mean abundance.

## Discussion

Studies of aggregation are often snapshots of parasite distributions on hosts, while aggregation is expected to result from dynamic processes that vary over space and time. Our results show that despite this expected dynamic, aggregation in this host system is fairly parasite species-specific and predictable across years. Parasite species account for an important source of variation in aggregation scores, even after accounting for relationships between Poulin’s *D* and mean abundance (itself correlated with prevalence). Our results contrast those of previous studies wherein alternative measures of aggregation showed too much variation to consider parasite aggregation a characteristic of the infecting species (Boag et al., 2001). Observed relationships between aggregation and mean abundance were indeed negative, as predicted (Poulin, 1993; Shaw, Grenfell & Dobson, 1998), and were consistent across diverse species of ectoparasites; however, the predicted inverse relationship was not observed among the four endoparasites. Variation in aggregation and its relation to mean abundance can be due to statistical and biological drivers likely differing between associations. We discuss both statistical and biological factors known or suspected of influencing aggregation in ptarmigan parasites, and provide direction for future research on degree of aggregation and its relations to mean abundance.

Broadly speaking, parasite taxon (*i.e.,* insects, mites, coccidians, and nematodes) was not predictive of aggregation. Mean values of Poulin’s *D* were widely ranging within taxonomic groups and the relationship between aggregation and mean abundance did not differ meaningfully among the groups. We selected four taxonomic groupings to maintain statistical power given our sample size, while recognizing the taxon-based similarities and differences in parasite biology (*e.g.*, life histories are similar among lice, but differ from the other insect species included in comparisons, *i.e.,* the louse fly). Alternative groupings could have been included in models and might have accounted for variation in degree or patterning of aggregation. Considering more groups, however, would have led to too few species within each group for a meaningful analysis. Consideration of the ecto-/endosymbiont dichotomy provided a higher-level categorization, but at lower resolution. The difference between ecto- and endosymbionts in relationships between aggregation and sample mean abundance requires explanation. However, we first entertain other possible determinants of aggregation, both in this and other systems.

For example, the degree of damage caused by the symbiont might be predictive of aggregation. An increasingly aggregated distribution of virulent parasites among hosts increases the stability of the host-parasite system—as the consequences of intensity-dependent effects are limited to relatively few host individuals (Anderson & May, 1978; Wilson et al., 2002)—while also reducing the probability of multiple virulent parasite co-infections in individual hosts (Dobson & Roberts, 1994; Morrill, Dargent & Forbes, 2017). In contrast, decreased aggregation may be expected if intensity-dependent host mortality removes individuals with higher-intensity infections from the sample (Anderson & Gordon, 1982; Johnson & Wilber, 2017). Relevantly, many of the host-associated symbionts in this system that are considered comparatively benign in terms of their effects on ptarmigan hosts (*G. lagopi*, *L. affinis*, *O. chloropus*, and *T. lagopi*) demonstrated the lowest average degrees of aggregation, while the more pathogenic species were more aggregated (Stenkewitz et al., 2016; Stenkewitz, 2017). The pattern was not perfect, however: the relatively nonpathogenic *S. holoaspis* and *M. lagopus* both demonstrated relatively high aggregation (Stenkewitz, 2017). Given that degree of pathogenicity was not easily quantified, we could not include it in our models as a relevant predictor of aggregation. This potential relationship merits further study; virulence was an important consideration in evaluating aggregation levels in amphibian parasites (Johnson & Wilber, 2017).

That broad taxonomy was not predictive of aggregation is somewhat consistent with the findings of Poulin (2013). In that study, residuals from a log-variance—log-mean abundance regression were interpreted as measures of relative aggregation after the expected underlying variance-mean relationship was accounted for (*e.g.*, higher variance than predicted for a certain mean abundance corresponding to higher aggregation). Although broad taxonomy showed no significant effects in Poulin’s (2013) study, those models demonstrated a notable degree of variation in aggregation as being explained by parasite species, again in agreement with our results. However, we also demonstrated that additional factors relating to the biology of the parasite-host association—namely, whether the symbiont is an ecto- or an endoparasite—is an important predictor of aggregation, by means of the interaction with an underlying effect of sample mean abundance (*cf.* Poulin, 2013).

Previous research describes and predicts negative relationships between aggregation and prevalence, or mean abundance, which themselves are positively correlated (Poulin, 1993; Shaw, Grenfell & Dobson, 1998; Johnson & Wilber, 2017). Some explanations for these relationships are statistical; for example, aggregation may decrease with prevalence as more prevalent parasites are more “spread out,” and fewer hosts in a sample remain uninfected (Poulin, 1993). Other explanations are biological in nature; for example, with higher resistance across hosts, prevalence of infection decreases in a sample, but the intensity of infection among those low-resistance individuals included in samples may be magnified, thereby increasing aggregation (Eppert et al., 2002). In such a case, the negative prevalence- or mean abundance-aggregation relationship may reflect variation in frequency of hosts showing resistance across samples (Eppert et al., 2002). On the other hand, hosts with generally higher tolerance to infection may experience more prevalent and abundant, though possibly less variable, levels of parasitism. Variation in average host tolerance across samples may then also relate to negative aggregation-mean abundance relationships.

Whereas there are statistical explanations for negative relationships between mean abundance and Poulin’s *D*, we suspect that additional biological factors influence the actual relationships observed in this study. The negative relationships appeared specific to ectoparasites. Two non-mutually exclusive biological factors that need to be considered as possible explanations for this overall pattern are niche capacity and intensity-dependent transmission of parasites between hosts. If one assumes a theoretical maximum intensity of infection that any host may experience for a specific parasite species (niche capacity), then one must also accept a constraint on how mean abundance can change without affecting aggregation. Once the parasite’s niche capacity is approached on a host or hosts, mean abundance can further increase only by adding parasites to less infected or to uninfected hosts, with a consequent decrease in aggregation and increase in prevalence. In other words, when comparing samples with varying mean abundance, we should expect the negative relationship with aggregation whenever infection intensities in sufficient numbers of sampled host individuals approaches its maximum or niche capacity. Alternatively, if a parasite is horizontally transmitted, and especially if such transmission increases in an intensity-dependent way, then increases in abundance might either reflect or drive increased transmission between hosts in a sample, with related decreases in aggregation. These hypothetical causes of the mean abundance—aggregation relationship may also explain why the strong negative relationship was observed among ectoparasites in our sample; in brief, statistical and biological forces are operating in tandem.

In comparison, the option of horizontal transmission is absent for the endoparasites considered in this study. For most of those species, the theoretical upper limit on abundance is much higher, meaning there is less of a constraint promoting less aggregated distributions of parasites when there is higher mean abundance. The coccidians, *E. muta* and *E. rjupa*, both were recorded with average intensities between 200–300, but with maximum intensities >14,000. It is unlikely that many hosts were experiencing close to their theoretical maximum eimeriid load. *T. tenuis* is a small nematode that can infect at high intensities, with mean infection levels observed to be 10, or even 100 times higher in other bird host associations than the overall average observed in this study (Hudson, Newborn & Dobson, 1992; Hudson & Dobson, 1997). *T. tenuis* abundance too, then, is likely not often limited by niche capacity constraints in this ptarmigan population. For the eimeriids and this nematode, mean abundance can increase by addition of parasites to already heavily-infected individuals without decreases in aggregation; indeed, for the two eimeriids, within-host birth processes during their endogenous phase could increase mean abundance and aggregation simultaneously through the addition of parasites to previously-infected hosts (Grear & Hudson, 2011). The other nematode, *C. caudinflata*, is relatively larger and may be expected to be more constrained by niche capacity limitations. Were it considered alone, a negative relationship between mean abundance and aggregation of *C. caudinflata* might have been detected (see Fig. 1). Regardless, our model predicted mean aggregation levels that were not incompatible with the observed *C. caudinflata* values of *D*. We nonetheless caution that larger samples within each species may have allowed the emergence of species-specific (rather than ecto-/endoparasite-specific) relationships with mean abundance.

Assuming the ecto-/endoparasite dichotomy with respect to relationships between aggregation and mean abundance is real, we should consider broad differences in traits between these groups of parasites other than the leading explanations of differences in niche capacity and density-dependent transmission. Transmission of ectoparasites between hosts is expected particularly during brood-rearing and crêche formation (Nielsen et al., 2020) and is expected to be mediated by the louse fly for certain parasites (Morrill et al., 2021). One broad difference between parasite types concerns variation in factors affecting the viability or availability of infective stages. Infective stages of both *T. tenuis* and the coccidians are likely to be affected by environmental conditions (*e.g.*, humidity). Transmission of *C. caudinflata* additionally requires an intermediate invertebrate host, the rainworm, the availability of which is dependent on wet conditions (Skírnisson, Thorarinsdottir & Nielsen, 2012). Aggregation levels of the two eimeriid species are also highly temporally dynamic (Þórarinsdóttir, Skírnisson & Nielsen, 2010). Variability in these factors might contribute additional “noise,” thus obscuring potential underlying relationships between aggregation and mean abundance among endoparasites. For ectoparasites, variation in the frequency and intensity of dust bathing which may help to remove oils and parasites from feathers (Montgomerie, Lyon & Holder, 2001) might contribute to variation in mean abundance or its relationship with aggregation.

In any discussions of determinants of aggregation, it is crucial to consider the analytical technique used. Beta regression provides an effective and alternative tool for examining predictors of parasite aggregation. Previous attempts to model biological predictors of parasite aggregation using other indices have been limited by the constraints on the potential variance of log-variance—log-mean relationship slopes, or the strong relationship between the aggregation index and sample mean abundance (*e.g.*, the variance-to-mean ratio, or the dispersion parameter *k* of the negative binomial distribution; Amarante et al., 2015; Cattadori, Boag & Hudson, 2008; Johnson & Wilber, 2017). Poulin’s *D,* which is a relatively common measure that aims to intuitively express what parasitologists commonly mean by “aggregation,” lends itself well to across-study analyses. In such research, values from a variety of host-parasite systems and studies can be combined into a single analysis using beta regression to search for general predictors of aggregation (*cf.* Poulin, 2013, where the log-variance–log-mean relationship was considered instead). The Bayesian approach would provide additional benefit here, as uncertainty in each observed *D* and differing degrees of replication across studies could be accounted for by having the central linear model describe the average actual, but unobserved, degree of aggregation. Here, variance derived from standard errors or confidence intervals around the reported *D* values could be incorporated; *i.e.,* together with the mean describing a distribution from which the observed values were “sampled.” This would effectively assign more weight in the model to observations with higher certainty (such an approach was not deemed necessary in our methods given the consistency in sample sizes). Alternatively, where uncertainties in reported *D* values were unknown, researchers could add a normally-distributed “noise” parameter to the linear model, the variance of which could potentially be inversely related to the number of replicates in each focal study. Using our described beta regression approach, researchers focusing on explaining other parasitological measures in addition to reporting aggregation can now extend their modeling of environmental and host-related predictors of those variables to aggregation, measured using Poulin’s *D*, rather than stopping their analyses after modeling predictors of measures like prevalence and abundance (Rodrıguez-Hernández et al., 2021; Xu et al., 2021).

A similar measure to *D*–the Hoover index (HI)–would also lend itself to modeling using beta regression. HI can be translated into the percentage of infecting parasites that would need to be “redistributed” among hosts to achieve an even distribution (McVinish & Lester, 2020). Modeling predictors of HI could provide more interpretable estimated model parameters (*e.g.*, a doubling of mean abundance for parasite species A would result in X% fewer parasite individuals needing to be redistributed to achieve an even distribution). There are also limitations to modeling aggregation using beta regression: *D* (or HI) must be measured consistently at a given sample level to allow comparisons across models (in our study, *via* ELPD). In the present study, for example, we could not look at the relationship between host age or sex and aggregation for each parasite species, because such stratification would result in different numbers of samples across models (one sample per species per year, *vs.* two or four if one or both host-level variables were also considered). We could not address whether any potential associations between co-infecting parasites explained patterns of aggregation, as such modeling would necessarily apply exclusively to individual host-level data; however, we do not expect co-infection to impact aggregation in this system as previous research demonstrated that any associations between these parasites explained on average only 2.01% of the observed variation in abundance, and never more than 5.13% (Morrill et al., 2021). To answer those questions regarding host-level predictors of aggregation or co-infection, an alternative modeling approach with a multivariate response could be used that considers parasite abundances for each species as following, for example, negative binomial (Poisson-Gamma mixture) distributions, that then additionally estimates the distributions’ dispersion parameters. This strategy could also incorporate a latent variable approach to determining how the abundances of co-occurring parasites are correlated after taking other predictors into account, in a similar fashion to some recent Bayesian implementations of joint species distribution modeling (Hui, 2016; Ovaskainen et al., 2017). Notably, while generalized linear models with a negative binomial response have been applied to describe parasite abundances for many years (Wilson, Grenfell & Shaw, 1996), doing so with the goal of estimating predictors of aggregation would now be possible in a Bayesian framework using hierarchical modeling (*e.g.*, a Poisson-Gamma GLMM with an indexed dispersion parameter), yet this was considered impossible by researchers in previous decades given technological limitations (Shaw, Grenfell & Dobson, 1998). Any approach to modeling abundances directly would, however, be less applicable to modeling aggregation across different studies (without access to the original raw data), an application we consider particularly apt for beta regression applied to Poulin’s *D*.

## Conclusions

Parasite aggregation reflects a balance of aggregative and disaggregative mechanisms operating in nature. Whereas observational studies of patterns of aggregation cannot in themselves distinguish between causes of (dis)aggregation, they can nonetheless suggest the level at which explanations are required. We found that parasite species was an important predictor of aggregation for this assemblage of ptarmigan parasites, and that aggregation and mean abundance were inversely related among ectoparasites, but not endoparasites. Future work should look at whether the ecto-/endoparasite dichotomy is generalizable using beta regression. Future research also could address how frequent proposed mechanisms promoting or reducing aggregation are active in host-parasite associations, and what levels of aggregation they produce when operating. The generality of our findings regarding species-level consistency in aggregation must be explored further as well; how much of the parameter space of Poulin’s *D* is generally occupied, and how consistent are species associations in showing a particular degree of aggregation in any given host system? We expect associations with the highest levels of aggregation to have at least one aggregative mechanism operating unchecked. Finally, *a priori* considerations of aggregation should be extended to macrosymbionts other than parasites, as some putative mechanisms of aggregation are parasite specific.

##  Supplemental Information

10.7717/peerj.13763/supp-1 Supplemental Information 1 R script for importing and cleaning the raw data, and fitting the model This R script requires that the raw data first be downloaded from the Dryad repository, that the Stan code file for the best-fitting model (also in supplementary material) is available, and that CmdStan is installed.

10.7717/peerj.13763/supp-2 Supplemental Information 2 Prior predictive simulations of the selected intercept and slope priors

10.7717/peerj.13763/supp-3 Supplemental Information 3 Stan code for the best-fitting model The Stan code for the best-fitting beta regression model, as described in the text. The R script also included in the supplementary material fits this model to the raw data hosted on the Dryad repository.

## Additional Information and Declarations

### Data Availability

The following information was supplied regarding data availability: The data is available at Dryad: Nielsen, Ólafur et al. (2020), Data from: Host sex and age typically explain variation in parasitism of Rock Ptarmigan: implications for identifying determinants of exposure and susceptibility, Dryad, Dataset, https://doi.org/10.5061/dryad.76hdr7stg. The Stan code for the best-fitting model, as well as an R script to fit the model using the data which is hosted on Dryad, is available in the Supplementary File.
