# Supplementary material for: Identifying sources of variation in parasite aggregation
Source: PeerJ. 2022 Aug 24;10:e13763. doi: 10.7717/peerj.13763 (PMC9419717; doi:10.7717/peerj.13763)
Supplement: Supplemental Information 2 [file peerj-10-13763-s002.pdf]

Identifying sources of variation in parasite aggregation

Morrill A, Nielsen ÓK, Skírnisson K, Forbes MR

Supplementary article S1: Prior predictive simulations

Intercept (θ) prior

As the linear model describes the average aggregation on the logit-transformed scale, the relatively uninformative normally-distributed θ prior is centred at zero (representing a value of 0.5 when back-transformed to the initial response scale). Given the expectation for aggregation among macroparasites, a more informative prior centred at a higher value could have been justified as well; regardless, our chosen prior still respected the realistic range of possible emergent aggregation levels while being somewhat skeptical of extreme values. The relatively low standard deviation value of 1.5 on the θ prior was necessary to keep the originally normally-distributed probability densities from becoming concentrated at zero and one when inverse-logit-transformed back to the response scale, and allowed a range of plausible D values across the permitted range, equally on either side of 0.5. Both wide and narrow beta distributions are plausible given the prior on the distribution variance parameter ($v \sim$ Half-Cauchy(2, 2.5)), as demonstrated in Figure S1 where 30 random beta distributions are simulated directly from the intercept (θ) and variance (v) priors (i.e. where the beta distribution mean, μ, is sampled directly from the intercept prior).

Slope (β) prior

The weakly informative prior for the slope parameter (β) was also normally distributed and centered at zero, with positive and negative effects of transformed sample mean abundance on aggregation therefore equally plausible. A standard deviation of 2.2 on the prior covered a wide variety of relationships from no relationship to strong relationships, while remaining skeptical of extreme correlations. Predictive simulations of 30 random trends based on samples from the intercept and slope priors, where trends are transformed back to the response scale (Poulin's D between zero and one), are presented in Figure S2. The simulated trends effectively cover a wide range of plausible relationships (weak, relatively strong, or none) across the observed range of sample mean abundances.

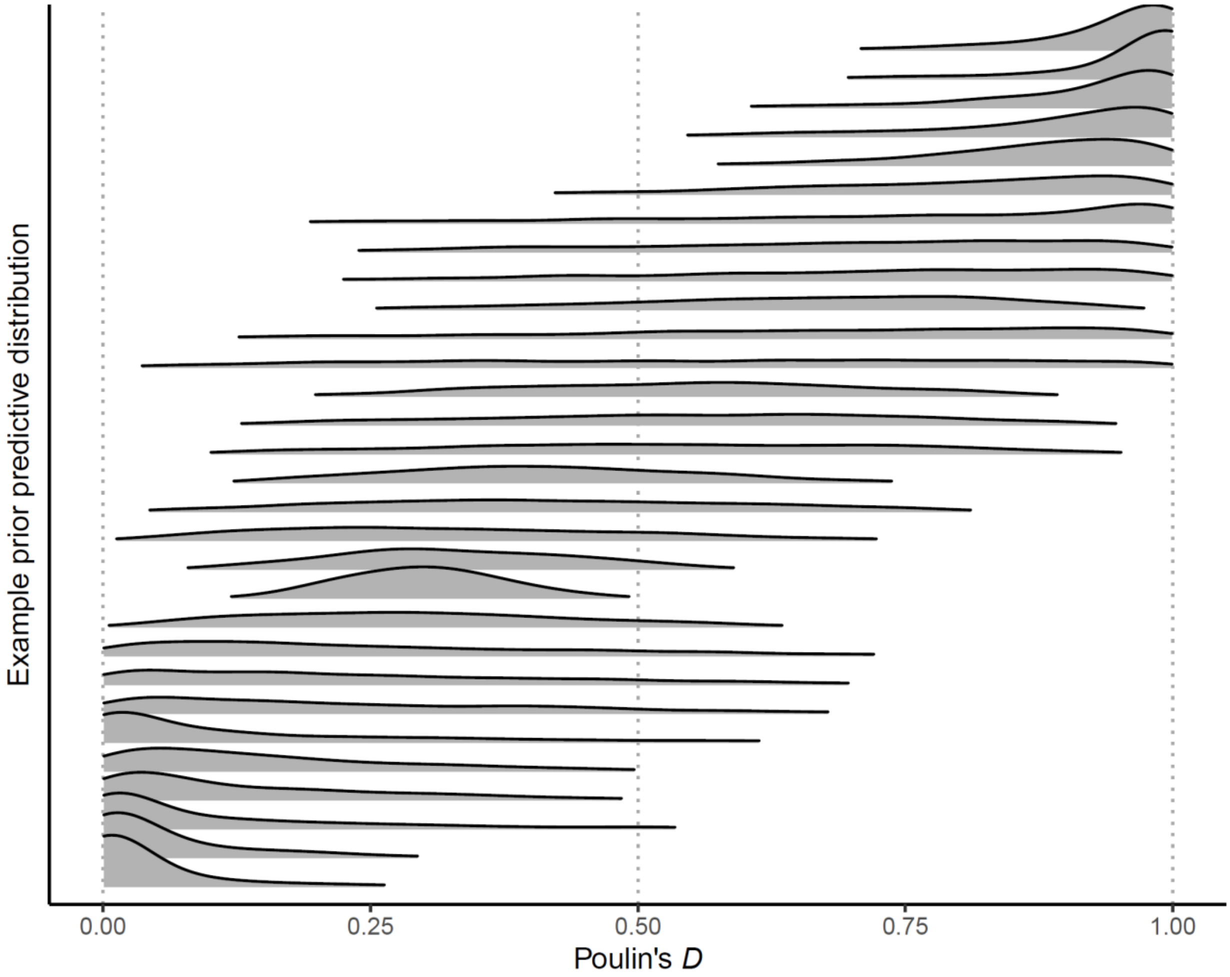

Figure S1: Density distributions from prior predictive simulation of 30 random beta distributions, with means sampled from the chosen intercept prior (θ ~ Normal(0, 1.5)) and transformed back to the response scale, and variance prior (ν ~ Half-Cauchy(2, 2.5). Distributions are ordered from top to bottom according to their means.

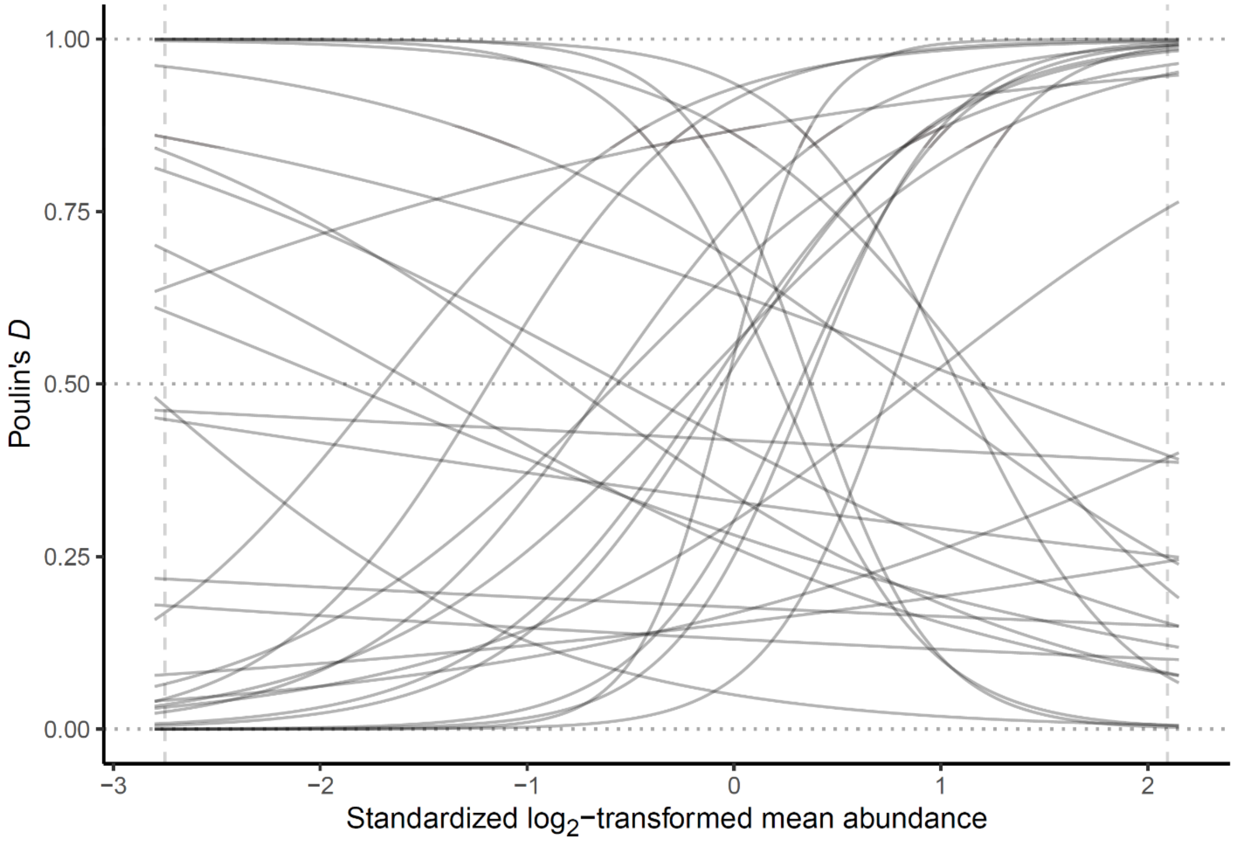

Figure S2: Simulated trends based on 30 samples from the intercept ($\theta \sim$ Normal(0, 1.5)) and slope priors ($\beta \sim$ Normal(0, 2.2)), based on the considered linear model relating mean abundance to aggregation, transformed back to the response scale. Dashed vertical lines represent the minimum and maximum sample mean abundances observed in the data.
